# Prospects of potential adipokines as therapeutic agents in obesity-linked atherogenic dyslipidemia and insulin resistance

**DOI:** 10.1186/s43044-023-00352-7

**Published:** 2023-04-04

**Authors:** Probin Kr Roy, Johirul Islam, Hauzel Lalhlenmawia

**Affiliations:** 1 Department of Pharmacy, Regional Institute of Paramedical and Nursing Sciences (RIPANS), Aizawl, Mizoram 796017 India; 2 Coromandel International Limited, Hyderabad, Telangana 500101 India

**Keywords:** Adipokines, Adipose tissue dysfunction, Anti-inflammatory adipokines, Atherogenic dyslipidemia, Insulin resistance, Metabolic disorder

## Abstract

**Background:**

In normal circumstances, AT secretes anti-inflammatory adipokines (AAKs) which regulates lipid metabolism, insulin sensitivity, vascular hemostasis, and angiogenesis. However, during obesity AT dysfunction occurs and leads to microvascular imbalance and secretes several pro-inflammatory adipokines (PAKs), thereby favoring atherogenic dyslipidemia and insulin resistance. Literature suggests decreased levels of circulating AAKs and increased levels of PAKs in obesity-linked disorders. Importantly, AAKs have been reported to play a vital role in obesity-linked metabolic disorders mainly insulin resistance, type-2 diabetes mellitus and coronary heart diseases. Interestingly, AAKs counteract the microvascular imbalance in AT and exert cardioprotection via several signaling pathways such as PI3-AKT/PKB pathway. Although literature reviews have presented a number of investigations detailing specific pathways involved in obesity-linked disorders, literature concerning AT dysfunction and AAKs remains sketchy. In view of the above, in the present contribution an effort has been made to provide an insight on the AT dysfunction and role of AAKs in modulating the obesity and obesity-linked atherogenesis and insulin resistance.

**Main body:**

“Obesity-linked insulin resistance”, “obesity-linked cardiometabolic disease”, “anti-inflammatory adipokines”, “pro-inflammatory adipokines”, “adipose tissue dysfunction” and “obesity-linked microvascular dysfunction” are the keywords used for searching article. Google scholar, Google, Pubmed and Scopus were used as search engines for the articles.

**Conclusions:**

This review offers an overview on the pathophysiology of obesity, management of obesity-linked disorders, and areas in need of attention such as novel therapeutic adipokines and their possible future perspectives as therapeutic agents.

**Graphical Abstract:**

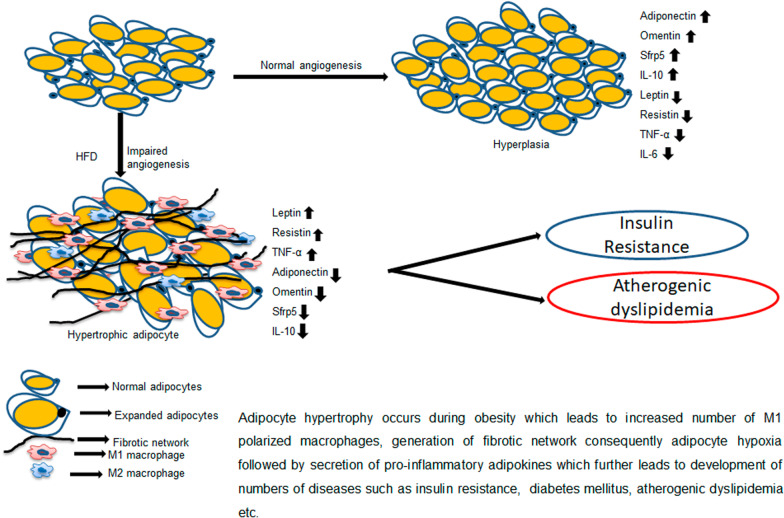

## Background

The outrage of obesity and its metabolic disorders is a major problem worldwide [1], and it is the cause of a higher premature death rate [2]. World Health Organization (WHO) estimated over 1.9 billion adults and older are overweight, out of which 650 million adults were obese in 2016. It is estimated that about 13% of the total world’s adult populations (11% men and 15% of women) were reported to be obese in 2016. The prevalence of obesity had tripled between 1975 and 2016 [3]. Obesity has a devastating effect on the vascular system creating adverse conditions that favor coronary artery disease (CAD). During obese state, the risk of various microvascular diseases such as hypertension, atherosclerosis, and myocardial infarction (MI) increases dramatically [4] and has been declared a major cause of death in both developed and developing nations in the twenty-first century [5]. Childhood obesity is one of the alarming concerns putting children and adolescents in poor health risk. As per the Centers for Disease Control and Prevention (CDC), the prevalence of obesity was 19.3% and affected about 14.4 million children and adolescents in the USA. Obesity prevalence was 13.4% among 2- to 5-year-olds, 20.3% among 6- to 11-year-olds, and 21.2% among 12- to 19-year-olds [6]. Therefore, obesity is not only a health hazard for the elderly but also children. Adipose tissue (AT) plays a vital role in the development of inflammation that contributes to the development of cardiometabolic risks in obesity [7, 8]. Abdominal obesity is one of the primary risk factors which is associated with blood-lipid disorders, inflammation, insulin resistance or type 2 diabetes mellitus (T2DM), thereby increasing cardiovascular morbidity [9]. Persons having abdominal obesity or with a central deposition of AT are highly susceptible to cardiovascular morbidity and mortality, including stroke, congestive heart failure and MI [10, 11]. Adipokines are generally produced by AT and involve different mechanisms such as energy homeostasis, metabolism, thermogenesis, reproduction, and immunity [12]. There are two different types of adipokine produced by fat tissue. The pro-inflammatory adipokines (PAKs) include resistin, leptin, tumor necrosis factor α (TNF-α), etc., are produced in higher quantity during obese state. The anti*-*inflammatory adipokines *(*AAKs*)* are adiponectin, omentin-1, secreted frizzled-related protein 5 (Sfrp5), and a few members of C1q/TNF-related protein (CTRP) family. These adipokines have a close link to inflammation and cardiovascular health via paracrine effects or by affecting endothelial function [12, 13]. During obesity, expression of PAKs is upregulated while of AAKs is downregulated. The presence of higher levels of AAKs is presumed to have protective action against obesity and associated damage and may play a crucial role in the management of obesity-linked cardiometabolic complications. Therefore, in this review we offer an overview on the pathophysiology of obesity, management of obesity-linked disorders, and areas in need of attention such as novel therapeutic adipokines and their future perspectives.

## Main text

### Microvascular dysfunction in adipose tissue during obesity

AT undergoes several biochemical changes that are involved in pathophysiology in the development of cardiometabolic disease (CMD). AT is known as the biological reservoir of energy (caloric). Adipocytes are the primary cell type responsible for the storage of excess calorie as triglyceride (TG) in the cellular lipid droplet without causing lipotoxicity to other cells. They expand to accommodate TG within the adipocyte [14].

#### Effects of expansion of fat in the microvascular system of adipose tissue

AT is composed of adipocytes, and other cell types, such as lymphocytes, macrophages, fibroblasts, and vascular cells [8]. AT expands and stores lipids in response to chronic excess caloric conditions [15], playing a vital role in appropriate angiogenesis, vascular and extracellular matrix (ECM) remodeling [16]. AT expands through the combination of adipocyte hypertrophy of pre-existing cells and hyperplasia [17]. Adipocyte hyperplasia permits healthy expansion of AT, while adipocyte hypertrophy without hyperplasia leads to lipid overload, causing adipocyte dysfunctions, resulting in cell death, initiation of AT inflammation and dysfunction followed by number of steps which leads to the development of insulin resistance and atherogenic dyslipidemia [18].

In obesity, adipocyte size gets increased, but there is no such concomitant increase in microvascular capillary density. Therefore, the demand for critical nutrients such as oxygen, glucose, and lipids could not be fulfilled due to insufficient capillary density [19], and hence, a group of adipocytes is cut off from the main supply to the vasculature, and initiates inflammatory processes [20]**.** AT has dense microvessels to maintain the tissue perfusion and nutrient supply adequately. It is believed that responsiveness of these microvessels is altered during obesity thereby having a significant impact on metabolism as well as nutrient transfer leading to insufficient AT perfusion and resulting in AT hypoxia.

#### Immune cell infiltration in AT dysfunction

Hypertrophic adipocyte necrosis (HAN) is a consequence of AT expansion; HAN contributes to the infiltration of macrophages in AT [21], thereby increasing the numbers of T cells, B cells, macrophages, neutrophils, and the mast cells. Anti-inflammatory cytokines interleukin (IL)-10 and transforming growth factor beta (TGF-β) are also released by M2 macrophage and T regulatory cells (Treg), which increases the insulin sensitivity and inhibits AT inflammation and dysfunction [22]**.** In lean AT mass conditions, macrophages in AT express CD206 (CD206 +) but CD11c (CD11c-) are not expressed, whereas, in obese tissue macrophages express CD11c (CD11c +) but not CD206 (CD206-) [23]**.** CD11c + is also known as M1 polarized, and it is believed to be the contributor to inflammation and metabolic dysfunction of AT in obesity. Polarization of M1 increases the production of hypoxia-inducible factor 1α (HIF1-α) [24], which upregulates pro-inflammatory cytokines such as interleukin-6 (IL-6), tumor necrosis factor-α (TNF-α) and monocyte chemoattractant protein-1 (MCP-1). These cytokines damage the microvessels. Damages to the AT arterioles lead to the dysregulation of the AT microcirculation [24, 25].

Other mechanisms involved in the progression of AT inflammation are endoplasmic reticulum (ER) stress and oxidative stress. Obesity induces ER stress in AT and liver tissues. Nutrients such as lipids and cytokines trigger the inflammatory kinases, e.g., c-Jun amino-terminal kinase (JNK), nuclear factor kappa-β(NF-kβ), inhibitor of kinase-β (IKK-β) at the molecular and cellular levels [26]. During ER stress, a complex response called unfolded protein response (UPR) takes place to maintain the functional integrity of the organelles through three major signaling molecules namely inositol-requiring enzyme 1 (IRE-1), PKR-like endoplasmic reticulum kinase (PERK) and activating transcription factor 6 (ATF6) [27]. The presence of ER stress activates JNK and IKK, which regulates the production of inflammatory cytokines including TNF-α. Exposure to TNF-α induces ER stress, and ER stress itself increases the expression of TNF-α resulting in more general inflammatory responses [28]. Similarly, reactive oxygen species (ROS) emerges from the mitochondria and/or ER and activates JNK and IKK, eventually, more ER stress, blocks insulin action and produces more ROS and causes broader inflammatory responses due to oxidative stress. The outcomes of oxidative stress in metabolic diseases are directly linked to diabetic complications through endothelial dysfunction [29]. In oxidative stress and insulin resistance, inflammatory pathways such as NF-kβ and JNK are activated in adipocytes, muscle cells, and impair insulin secretion in pancreatic β-cells [30]. In T2DM, β-cells synthesize and secrete insulin continuously due to its activation associated with unresolved hyperglycemia, thereby causing cellular stress that induces deterioration and apoptosis of β-cells [31].

In the obese state, the number of adipose tissue macrophages (ATMs) present in AT plays a critical role in the progression of metabolic dysfunction. ER stress has been shown to suppress M2 polarization of macrophages in obesity [32]. M2 macrophages usually generate anti-inflammatory cytokines IL-10 and IL-1 decoy receptors. M2 polarization results in increased production of “arginase”, an enzyme which blocks inducible nitric oxide synthase (iNOS) activity and competes with the arginine, a substrate required for nitric oxide (NO) production [33]. M2 polarization occurs via activation of Signal Transducer and Activator of Transcription 3 (STAT3) and STAT6 pathways by IL-4/13 and IL-10 secreted by T helper 2 (TH2) cells. On the other hand during ER stress, pro-inflammatory cytokines such as IFN-γ, TNF-α or Toll-like receptors (TLR) are released resulting in M1 polarization. AT is dominated by M1 macrophages and inflammatory pathways like NF-kβ and STAT1 are activated which suppresses the M2 polarization and resulting production of pro-inflammatory cytokines such as TNF-α, IL-6, IL-1β and consequently AT inflammation [34].

In AT dysfunction, M1 macrophages form aggregates around the necrotic lipid droplets that are formed as a result of adipocyte lipolysis [35]. After adipocyte lipolysis, the leukocyte aggregates are shared with mast cells, CD4 + and CD8 + T cells. In AT, CD4 + T_H_ cells include Treg, TH1, and TH2 and CD8 + T regulates local inflammation through the cytokine secretion which is involved in the differentiation and polarization of macrophages [36]. Polarization of M1 macrophages stimulates the inflammatory cytokine production and increased infiltration of pro-inflammatory CD8 + T and shifts towards higher CD8 + T/CD4 + ratio [36]. In this condition, infiltration and accumulation of T cells (CD8 + , and TH1 CD4 + T) leads to loss of Treg anti-inflammatory cells followed by induction of B cells, natural killer (NK) cells, Type-1 natural killer (NKT) cells, eosinophils, neutrophils, and mast cells [37]. These cells helps in the progress of atherosclerotic progression through the release of pro-inflammatory cytokines including TNF-α, leptin, IL-6, resistin, etc. M1 macrophages are immunoreactive to oxidized low density lipids (oxLDL) resulting from lipolysis in adipocytes. The accumulation and retention of LDL within the artery walls is mediated by interaction between apolipoprotein B-100 and proteoglycan binding and undergoes oxidation and enzymatic modification and produces oxLDL [38]. Accumulation of oxLDL triggers inflammatory response and activates cells within arterial intima and induces the expression of inflammatory cytokines, chemokines and adhesion molecules. The adhesion molecules then adhere monocytes to endothelium and migrate to arterial intima [39]. Failure to remove accumulated oxLDL by scavenger receptors results in cholesterol droplets available to cytosol and transform these macrophages into foam cells, an early characteristic of atherosclerosis [40].

Fatty acid metabolism is regulated by peroxisome proliferator-activated receptor (PPAR) and liver X receptor (LXR). These two regulate fatty acid metabolism transcriptionally. PPAR controls fatty acid degradation, whereas LXR regulates the synthesis of fatty acid by activating sterol regulatory element-binding protein-1c [41]. Despite their opposite action in lipid metabolism, PPAR and LXR enjoy some common features and have anti-atherosclerotic effects. PPAR controls the cholesterol efflux in foam cell macrophages through the LXR-dependent ATP-binding cassette (ABC) pathway and activation of PPAR inhibitors foam cell formation and thereby atherosclerosis [42, 43]. Activation of the LXR upregulates the expression of ABCA1 and ABCG1 and accelerates reverse transport of cholesterol [44]. Activation of LXR also increases the expression of ABCG5 and ABCG8 in the intestine tissue, which regulates the absorption of cholesterol and protects against atherosclerosis [45]. Similar action is seen with PPAR activation in rats and mice [46]. Both LXR and PPAR facilitate the movement of cholesterol from peripheral cells to the feces and are called reverse transport cholesterol.

In obesity, oxLDL is recognized by toll-like receptor-4 (TLR-4) and plays a critical role in development of atherogenesis. Activation of TLR-4 enhances lipid uptake by macrophage thus develops foam cells [47]. Polarized M1 stimulates TLR-3, TLR-4 or TLR-9 and upregulates the expression of scavenger receptor A, macrophage receptor with collagenous structure (MARCO) and lectin like low-density lipoprotein receptor-1 (LOX-1), hence enhancing foam cell formation [48].

#### Role of obesity in alteration of vascular structure and function of AT

The link between obesity and vascular endothelial growth factors (VEGF) is crucial in the development of hypertension and atherosclerosis [8]**.** During obesity, VEGF secretion increases in an insulin-dependent manner [49]. VEGF levels also rise during the expansion of vascular adipose tissue (VAT) [50, 51]**.** VEGF-A improves vascularization and turns white adipose tissue (WAT) to brown adipose tissue (BAT). This is associated with an increase in energy expenditure and attenuates diet-induced metabolic effects such as insulin resistance and hepatic steatosis [51, 52]**.** On the contrary, in obesity, adipocytes restrict deletion of VEGF-A resulting in limited AT vascularization thereby higher AT inflammation and systemic metabolic dysfunction [4, 53]. HIF1-α is the key regulator of VEGF expression, which gets upregulated in AT expansion during obesity [53].

Fat expansion outgrows the blood supply due to deficient angiogenesis and prompt ischemia, hypoxia, necrosis, and inflammation within the adipose milieu [54]**.** The individuals with obesity develop capillary dropout and suffer a deficiency of vascularization, mainly in visceral fat; the ensuing consequences are inflammation and metabolic dysfunction [24, 55]**.** A marked difference is also observed in genetic transcription of visceral fat and subcutaneous fat in the obese state in comparison to lean state [24]**.** For instance a gene Angiopoietin-like 4 (ANGPTL4)is mainly expressed in AT [56]**,** secreted by adipocytes and is known to possess pro-angiogenic effect and has been studied thoroughly due to its inhibitory effect on lipoprotein lipase, an enzyme which is responsible for TG metabolism, and responsible for the triglyceridemia when overexpressed [57, 58]**.**

Circulating leukocyte recruitment in the endothelium represents the pathophysiology of macrovascular and microvascular diseases [59]. Under normal circumstances, endothelium does not bind/interact with circulating leukocytes. Various adhesion molecules including selectins and cellular adhesion molecules (CAMs) are expressed in the luminal surface of endothelial cell during the early stage of endothelial dysfunction and these molecules act as receptors for glycoconjugates and integrins which are present in the circulating leukocytes [60]. Traditionally, it has been believed that prolonged exposure of the vascular endothelium to elevated circulating levels of metabolites or inflammatory mediators, such as glucose, free fatty acids (FFAs), oxLDL, and cytokines, and endothelial dysfunction occurs by perturbing endothelial cell homeostasis [61]. However, as the research progressed over the periods of time, recent research emphasizes the role of AT and unbalanced secretion of mediators by adipocytes in obesity as major causes of endothelial dysfunction [62]. AT dysfunction leads to the activation of inflammatory signals that directly or indirectly act from white adipocytes and actively contributes to the circulating milieu and induces vascular dysfunction [63].

Under normal physiologic conditions, the type I transmembrane glycoprotein vascular cell adhesion molecule-1 (VCAM-1) expression is absent or very low, however, its expression can be triggered by cytokines such as TNF-α [60] and the role of VCAM-1 on atherosclerosis is well explained in animal as well as in human study[64, 65]. Apart from CAM expression, endothelium dysfunction causes loss of endothelial NO (eNO). Consequences of loss eNO are hypertension to several associated complications, including increased endothelial adhesion molecules expression which further leads to development of atherosclerosis [66]. NO possess anti-inflammatory effect and the effect is mainly based on the inhibition of the leukocyte–endothelial interactions. NO exert the anti-inflammatory effect by inhibiting exocytosis of Weibel Palade bodies and reducing NF-kβ expression [67].

Endothelial dysfunction is an early marker of cardiovascular disease (CVD), healthy endothelium is actively capable of inhibiting the pro-atherogenic process by NO pathway. AT express numbers of PAK including leptin, resistin, TNF-α, as well as AAK including adiponectin, Sfrp5, CTRPs, etc., respectively. ATMs are responsible for the production of these adipokines. Adhesion molecules such as P-selectin, E-selectin, and intracellular adhesion molecule (ICAM-1) are highly expressed in AT. Leukocyte recruitment, rolling and MCP-1 are increased with the adhesion molecule expression and promotes leukocyte transmigration and integrins, which increases the adherences to the intima [7]. In this condition phagocytosis of LDL particles by monocytes leads to formation of foam cells and develops a fatty streak followed by plaques. These plaques are very prone to rupture followed by thrombus formation which subsequently favors the occlusion of artery and infarction occurs. PAK modulates smooth muscle cell constriction, proliferation and migration. PAK also hampers the release of AAK from AT [68, 69]. TNF-α, IL-6 inhibits the expression and release of AAKs. PAKs like leptin, at high concentration, promote adhesion and transmigration of monocytes through the derived capillary endothelial cells (AT-ECs) [70]. Leptin upregulates the expression of MCP-1 and increases the production of endothelial ROS and JNK activity and also enhances the DNA binding activities of redox-sensitive transcription factors NF-kβ and activator protein-1(AP-1) [71]. Resistin also directly injures endothelium by increasing production and expression of adhesion molecule VCAM-1 and MCP-1 via endothelin-1 by endothelial cells [72]. During endothelial dysfunction circulating levels of AAKs are decreased. AAKs, e.g., adiponectin, exert anti-inflammatory effect on endothelial cells and inhibit TNF-α thereby reducing the expression of adhesion molecules and other inflammatory cytokines [73]. Therefore, the balance between AAKs and PAKs plays an important role in the development and progression of atherosclerosis.

Another most important harmful effect of obesity is arterial stiffness. Arterial stiffness is structural and functional changes in the intimal, medial and adventitial layers of the vasculature. In stiff arteries, the propagation of pulse wave is faster and due to increased velocity, an altered hemodynamic changes especially increased central systolic blood pressure and pulse pressure are observed which have an negative impact on myocardium due to increased left ventricular afterload and decreased coronary blood flow [74]. Arterial stiffness is considered one of the valuable risk factors for the CHD.

In obesity metabolic changes in AT result in altered secretion of hormones and cytokines such as TNF-α, IL-6, leptin, resistin, adiponectin, etc. Increased levels of adipocyte derived cytokines impairs the insulin sensitivity and enhances the recruitment and activation of pro-inflammatory immune cells in the vasculature which contribute in the development of arterial stiffness [75].

#### Obesity-induced fibrosis and remodeling of adipose tissue

Adipocytes in AT are encircled by ECM. ECM proteins provide mechanical support and regulate adipogenesis and lipid droplet growth. In the obese state, ECM undergoes modification to accommodate the adipocytes. In obesity, a rapid expansion of AT leads to ECM remodeling and thereby persistent hypoxia, which activates HIF1-α [76]**.** In obese state, there is 30–40% lower blood flow to AT, 44% lower capillary density and 58% lower VEGF growth [77]. Pre-adipocytes and mature adipocytes usually generate a substantial amount of macrophage migration inhibition factor (MIF). Expression levels of MIF are positively correlated with Body mass index (BMI) of an individual [78].

In obesity AT hypoxia leads macrophage infiltration to that hypoxic area of AT. Hypoxia activates macrophage, and subsequently activation of HIF1-α occurs which then inhibits differentiation of pre-adipocyte thus fibrosis of AT. Hypoxia also inhibits differentiation of adipocytes from pre-adipocytes [24]. Leptin signaling controls the inhibition of pre-adipocyte differentiation [79]. Pre-adipocyte shows higher expression of PAKs than the adipocytes. It is considered that, one per cent hypoxia is sufficient to enhance the significant release of VEGF, IL-6, and PAI-1 from pre-adipocytes; however, the hypoxic value stands for adipocyte is one and half of that of pre-adipocytes [80]. Under hypoxic conditions, adipocytes express HIF1-α and recruit HIF-1 protein [24]. Adiponectin and leptin secretion are very sensitive to hypoxic conditions of adipocytes. Hypoxia also modulates major inflammatory secretion of major inflammatory adipokines such as IL-6, MIF (macrophage migratory inhibitory factor), VEGF, serum amyloid A and matrix metallopeptidase 2 (MMP-2) and adiponectin [24]. Endotrophin, a compound generated during the cleavage of α3 subunit of collagen VI (COL 6), secreted by adipocytes promotes AT fibrosis and systemic metabolic dysfunction [81].

### Obesity-linked atherogenic dyslipidemia and insulin resistance

**Fig. 1:**
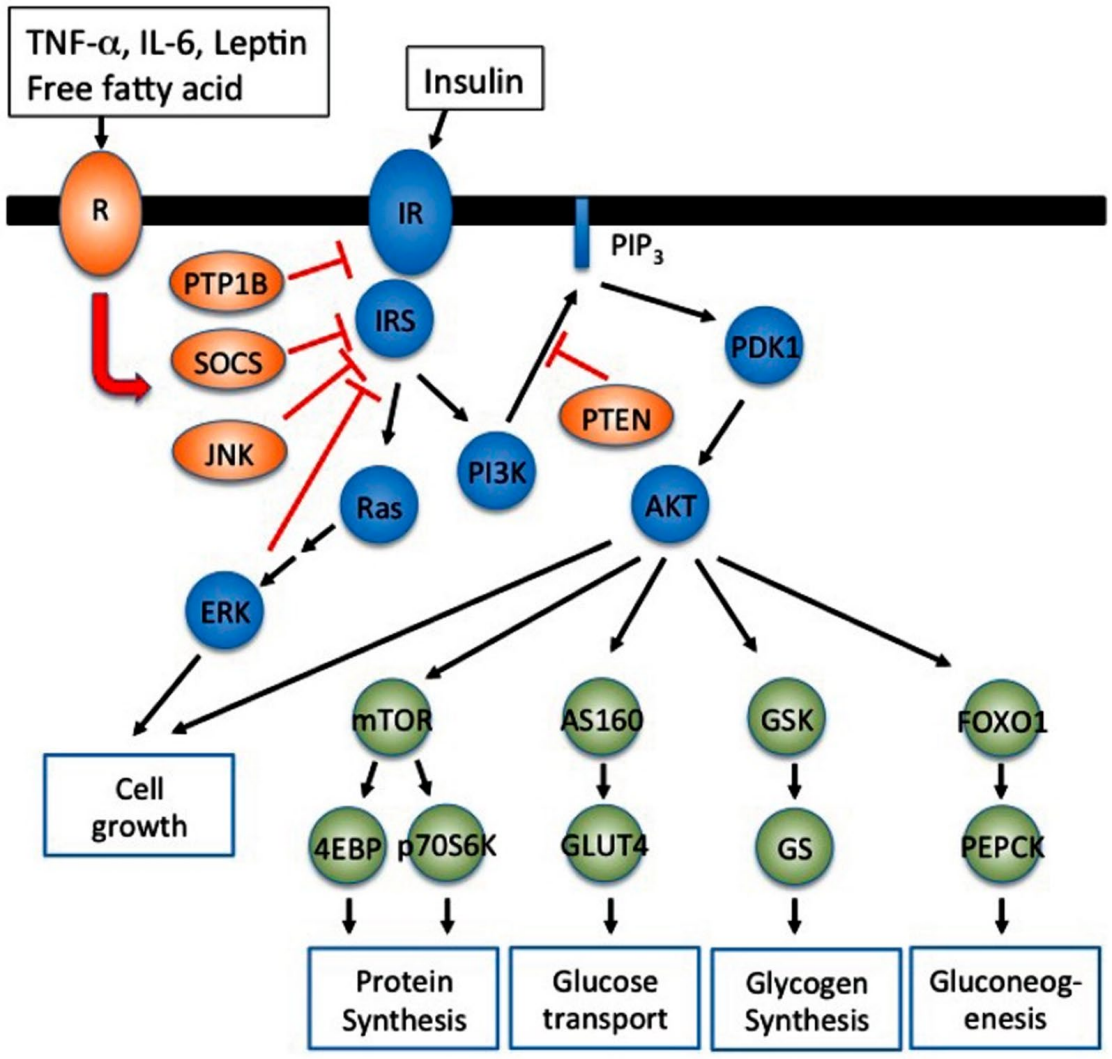
Inflammatory adipokines suppress insulin signaling resulting in insulin resistance. IRS1/2 phosphorylated on specific tyrosine residues activates the phosphatidyl inositol 3-kinase (PI3K)-AKT/protein kinase B (PKB) pathway and Ras-mitogen-activated protein kinase (MAPK) pathway. PI3K-AKT signaling pathway regulates metabolic processes such as glucose uptake(muscle and adipocytes), glycogen synthesis (muscle and liver), protein synthesis(muscle and liver), and gluconeogenesis (liver). Inflammatory signals, TNF-a, IL-6, Leptin and saturated free fatty acid, activate inhibitory molecules such as SOCS and JNK to suppress insulin signaling resulting in insulin resistance. PI3K-dependent PDK1 activation is negatively regulated by phospholipid phosphatases such as phosphatase and tensin homolog (PTEN) that degrade PIP3 [86]. doi: 10.3389/fendo.2013.00071, Reproduced with permission Frontiers in Endocrinology)

Atherogenic dyslipidemia and insulin resistance are the two main manifestations of CMD linked to obesity. The genetic component responsible for obesity and insulin resistance has not yet been completely understood. Vascular inflammation and diabetes are common phenomena in obesity [82]. Metabolic products like lipids, hormones, and cytokines formed as a result of obesity-related biochemical processes are also responsible for insulin resistance and metabolic dysfunction. Insulin resistance hinders the insulin signaling pathways in muscles, endothelial cells and AT [83]**.** The mechanisms started with PAKs or metabolic excess including TNF-α, endothelin-1, FFA or ER stress which exhibit ser/Thr phosphorylation of insulin receptor substrate 1(IRS1) and cause insulin resistance. Dysregulation of insulin signaling associated with numerous disorders such as dyslipidemia, hypertension, cardiovascular disease, stroke, etc. In insulin resistance, acute and chronic inflammation plays a dynamic role and also provides information about the role of diets, physiological stress and obesity. Inflammatory cytokines like IL-6, TNF-α stimulates lipolysis and generates free fatty acid from TGs during obesity. One of the main reasons for insulin resistance and T2DM is due to heterologous and feedback inhibition of insulin signaling which is mediated by phosphorylation of IRS1. Pro-inflammatory cytokines including IL-6 and TNF-α are produced from AT during obesity. TNF-α promotes serine phosphorylation of ISR1 and IRS2 and is closely associated with insulin resistance [26]. TNFα plays an active role in insulin resistance because of its ability to bind IRS1 and IRS2 thereby phosphorylates serine residue and inhibits insulin stimulated tyrosine phosphorylation [84]. Tyrosine phosphorylation at specific sites on receptor substrates are very important for glucose uptake, lipogenesis, and glycogen and protein synthesis, as well as for stimulation of cell growth [85]. Phosphorylation of serine residue of the insulin substrate interferes with the tyrosine phosphorylation by decreasing the binding of insulin receptors or degradation of IRS1(Fig. 1) [86].

Ubiquitin-mediated degradation of IRS1 and IRS2 is another mechanism which promote cytokine induced insulin resistance and have contribution in diabetes as well as in β cells dysfunctioning. Suppressor of cytokine signaling (SOCS) 1 and 3 are proteins which bind to distinct domains of insulin receptor and plays important role in insulin receptor mediated phosphorylation of IRS1 and IRS2. SOCS1 overexpression in the liver inhibits IRS2 tyrosine phosphorylation and SOCS3 overexpression decreases tyrosine phosphorylation in both IRS1 and IRS2 [87]. Resistin and leptin increase the expression of SOCS1/3 in liver which causes insulin resistance and upregulates the key regulator for the production of fatty acid synthesis and sterol regulatory element-binding protein 1c (SREBP-1c) expression. Thus, SOCS1 and SOCS3 are linked to inflammation, metabolic stress, insulin resistance and glucose intolerance.

Mitochondria is the major site of lipid degradation and plays an important role in metabolic health as mitochondrial dysfunction is associated with the ageing process as well as metabolic disorders [88].Maintenance of the intracellular redox environment (RE) is crucial in order to carry out cellular vital functions [89]. Mitochondria maintains intracellular RE and constitutes subcellular compartments with peroxisomes, the area for lipid degradation [90]. Fatty acids (FAs) are degraded by β-oxidation and its rate depends upon demand such as increased work and ATP utilization proceeds faster oxidative phosphorylation (OxPhos) and tricarboxylic acid (TCA) cycle activity.

Lipids are usually presented as albumin bound FAs by AT or by coronary vascular endothelial lipoprotein lipase as a catabolized very low density lipid (VLDL) complex. Long-chain FA (LCFA)transport occurs across sarcolemma through the carrier such as, fatty acid transporter protein 1(FATP1); plasma membrane-associated fatty acid-binding protein (FABP); long-chain fatty acid transporter (LCFAT); plasma membrane sodium-dependent carnitine transporter (OCTN2); fatty acid translocase CD36(FAT/CD36). Similarly in mitochondria, carnitine palmitoyltransferase 1(CPT1); carnitine acylcarnitine translocase (CACT).

LCFA when enters the cell, it forms thioesters with coenzyme A (CoA) and are oxidized in the mitochondria via β-oxidation or forms triacylglycerol (TAG) via esterification. TAG is stored in the form of lipid droplets. Activation of LCFA occurs by long-chain acyl-CoA synthetase in mitochondrial outer membrane. However, mitochondrial inner membrane limits the entry of acyl-CoAs. The transporter protein CPT1 plays an important role and converts long-chain acyl CoA to long-chain acylcarnitine, which is subsequently entered into the mitochondria [91].

A prominent theory states that the relation between the FA oxidation and insulin resistance. It suggests that muscle insulin resistance occurs due to the impaired mitochondrial uptake and fatty acid oxidation [92]. It explains that long-chain acyl-CoA derived from lipids or intramuscular triacylglycerol (IMTG) are diverted away from CPT1, the mitochondrial enzyme responsible for first and essential step in β-oxidation of LCFA. On the contrary, it is moved towards the synthesis of signaling intermediates such as diacylglycerol (DAG) and ceramide. Accumulation of these and other lipid molecules engaged stress activated serine kinases which interfere with insulin signal transduction[93, 94].

Dyslipidemia is a disorder in the contents of lipids, where cholesterol and TGs are the key factors that play a crucial role in the development of atherosclerosis. Atherogenic dyslipidemia is characterized by an elevated level of TG, and lower levels of high-density lipid cholesterol (HDL-C). The link between dyslipidemia, obesity and atherosclerosis have been studied thoroughly by many researchers. The formation of atherogenesis is influenced by diverse adipokines. Atherogenesis is not only about deposition of fat into the arterial wall but the role of the adaptive and innate immune system have to be considered [95]. Atherogenesis starts in the specific site where endothelium is submitted to shear stress clearly at aortic root, aortic arch, superior mesenteric artery, and renal arteries [96]. In this position, endothelial dysfunction and permeability of the intimal layer occurs which favors the migration of LDL particles to sub-endothelial space [97]. In the presence of leptin, TNF-α, endothelial dysfunction and transmigration of LDL particles get worse. Here, LDL particles are oxidized (oxLDL), which can be positively related to MCP-1 level. The presence of MCP-1, IL-6, leptin and TNF-α increases the expression of adhesion molecules such as VCAM-1 and ICAM-1 in endothelium and enhances leukocyte transmigration. Under the influence of MCP-1 monocytes are developed into macrophage and phagocytes oxLDL and turn into foam cells [98]. IL-6 is produced by smooth muscle cells (SMC) under the influence of angiotensin-II. IL-6 and MCP-1 increase the recruitment and proliferation of SMC and extracellular matrix to form a fibrous cap around the necrotic lipid core. In the presence of matrix metalloproteinases and prothrombotic molecules, MCP-1 and leptin help in rupturing the plaque formed and thrombus formation [96]. The atherosclerotic plaque thus formed causes occlusion of the coronary artery, thereby reducing the blood supply to the heart. Due to complete blockage of the coronary artery, the heart muscle does not get enough supply of oxygen and starts to die causing ischemia and eventually MI.

Although the treatment regime for the treatment of LDL cholesterol, blood pressure and glycemia have improved, atherogenic dyslipidemia remains as a silent killer due to being underdiagnosed and undertreated in clinical practice [99]. Atherogenic dyslipidemia is commonly associated with CVD, T2DM and contributes both macrovascular as well as microvascular residual risks. To reduce the residual risks of patients with atherogenic dyslipidemia, a residual risk reduction initiative was established to address this clinical issue. In 2014, a meeting with European experts in CVD and lipid was convened in Paris, France, to discuss atherogenic dyslipidemia, lipid and its associated CV risks. They concluded that elevated levels of LDL-c have greater risk for CV than low LDL-c and could be treated with statins. However, even after treating with statins some patients have abnormal lipid profiles especially with elevated levels of TGs, low levels of HDL-c which presents residual CV risk. Therefore, it was recommended to measure the levels of TGs and HDL-c to manage the overall residual CV risk. They recommended use of statin along with other lipid lowering drugs such as fenofibrate to achieve clinical benefits [100, 101]. Therefore, to counter atherogenic dyslipidemia along with proper diagnosis statin-combination therapy is recommended to get more clinical benefit patients with residual risk. However, this is not a proper treatment regime that can be completely safe and effective, therefore researchers focus on new drugs with more efficacy and ensuring the effectiveness is still awaiting in atherogenic dyslipidemia. Since adipokines levels change during dyslipidemia and AAKs have been reported to have anti-atherogenic effects, it would be interesting to see the adipokines' role as a marker and therapeutic agent in treating atherogenic dyslipidemia in the near future.

### Adipokines in atherogenic dyslipidemia and insulin resistance

Adipokines came to attention when the leptin, an AT specific adipokine, proved to be an important regulator for food intake and energy expenditure [102]. Since the discovery of leptin, new adipokine attracted the attention of researchers due to its utter responses between CVDs, obesity and metabolic disorder. This new adipokine plays numerous roles in the microcirculation of AT and affects target organs through autocrine, paracrine or endocrine pathways [103]. Adipokines are being classified according to their beneficial and harmful effect on the body. The beneficial effects of adipokines are cardioprotection, promoting endothelial function, angiogenesis, and insulin-sensitizing effect, whereas harmful effects include atherosclerosis, insulin resistance and inflammation [104]. The beneficial action of the adipokines are mostly exerted by AAKs, whereas PAKs are responsible for the deleterious effect. A list of preclinical and clinical studies of the AAKs are listed in Tables 1 and 2

**Table 1:** Preclinical evidence of anti-inflammatory adipokines in insulin resistance and atherogenic dyslipidemia

Adipokines	In-vitro model/in-vivo model	Administration mode	Action or application type	References
Adiponectin	Bovine aortic endothelial cells	–	Adiponectin has vascular action and stimulate the production of NO therefore causes vasodilation; possess anti-atherogenic properties	[105]
	Rabbit	Renal artery	Treatment with Adiponectin decreases the atherosclerotic plaque size	[106]
	Human aortic endothelial cells, human monocyte cell line	–	Adiponectin level is correlated with CAD risk	[107]
	Human aortic endothelial cells	–	Adiponectin modulates the inflammatory response of endothelial cells via NF-kβ signaling through a cAMP-dependent pathway	[108]
	Human umbilical vein endothelial cells	–	Protection of endothelial monolayer from angiotensin II, or TNF-induced hyper-permeability, modulation of microtubule and cytoskeleton stability via a cAMP/ PKA signaling cascade	[105]
	HUVECs	–	Suppression of endothelial cell apoptosis, vascular protective activities	[110]
	Ob/ob mice, ApoE-deficient Mice	–	Globular adiponectin (gAd) enhances fatty acid oxidation, ameliorate insulin resistance and atherosclerosis	[111]
	Ob mice, wild type mice	Subcutaneous injection	Adiponectin replacement therapy attenuates myocardial damage in leptin-deficient mice	[112]
	High-fat apolipoprotein E-deficient (ApoE − / −) mice	Via tail vein	Suppress oxidative stress, lipid production. Administration of adiponectin reduces atherosclerotic lesions formation size and rate in the aorta and reduces TC, TG, and LDL-c levels	[113]
	Rats	Tail vein injection	Adiponectin alleviate the coronary no-reflow injury in T2DM rats by protecting endothelium and improving microcirculation	[114]
	Adiponectin knockout mice or wild type mice	–	Adiponectin protects hearts from cardiac ischemia/reperfusion injury via inhibition of iNOS and nicotinamide adenine dinucleotide phosphate-oxidase protein expression and resultant oxidative/nitrative stress	[115]
	C57BL/6 mice	Intraperitoneal injection	Adiponectin activates AMPK pathway, regulates glucose metabolism and insulin sensitivity in vitro* and *in vivo	[116]
	. PPAR-γ + /– mice	Intraperitoneal injection	In insulin resistance the levels of adiponectin is decreased. Replenishment of adiponectin improves insulin sensitivity and diminishes diabetes	[117]
	Adiponectin-deficient (APN-KO) mice		Adiponectin protects the heart from ischemia–reperfusion injury via AMPK- and COX-2–dependent mechanisms	[118]
Omentin-1	Cardiomyocyte	–	In T2D, omentin-1 level is decreased and Omentin-1 act as cardioprotective adipokine	[119]
	Wistar rats	–	Omentin induces endothelium-dependent vaso-relaxation in rat isolated aorta via endothelium-derived NO through phosphorylation of eNOS	[120]
	Wistar Rats	–	Omentin -1 level is modulated by AT during diabetes. Increased omentin-1 level interferes with the glucose metabolism pathway by stimulating phosphorylation of Akt in muscle tissue	[121]
	Wistar rats, Cultured vascular smooth muscle cells	–	Omentin demonstrates anti-inflammatory effects, inhibits TNF-α induced VCAM. Omentin inhibits TNF-α-induced VCAM-1 expression via preventing the activation of p38 and JNK	[122]
	Wistar Rats	Subcutaneous	Omentin-1 reduces blood pressure in rats via production of NO. Other anti-inflammatory adipokines such as adiponectin is increased following omentin-1 administration	[123]
	Human Epicardial tissue	–	Circulating and epicardial AT-derived omentin-1 level decreased with patients with CAD	[124]
	Human monocyte-derived macrophages, human aortic smooth muscle cells (HASMCs), and aortic lesions of Apoe-/- mice		Omentin-1 promotes anti-inflammatory M2 phenotype during differentiation of human monocytes into macrophages Omentin-1 suppresses oxidized low-density lipoprotein-induced foam cell formation. Omentin-1 levels were markedly reduced in coronary endothelium and epicardial fat but increased in plasma and atheromatous plaques (macrophages/SMCs) in CAD patients compared with non-CAD patients	[125]
	Thoracic aortas of C57BL/6 mice	–	Omentin-1 reversed impaired endothelial-dependent relaxations (EDR) in mouse aortas. Omentin-1 treatment reverses elevated ER stress markers, oxidative stress and reduction of NO production. Omentin-1 protects against high glucose-induced vascular endothelial dysfunction through inhibiting ER stress and oxidative stress and increasing NO production via activation of AMPK/PPAR-δ pathway	[126]
	Apolipoprotein E-deficient (apoE-KO) mice	–	Omentin-1 act as anti-atherogenic adipokine that directly affects the phenotypes of macrophages Omentin reduces the development of atherosclerosis by reducing inflammatory response of macrophages through the Akt-dependent mechanisms	[127]
SFRP5	Human adipocytes and skeletal muscle cells (hSkMC)	–	Sfrp5 lowered IL-6 release and NF-κβ phosphorylation in cytokine-treated human adipocytes	[128]
	Mice	–	Sfrp5 have important roles in glucose regulation and β-cell function	[133]
	3T3‐L1 pre‐adipocytes	–	Sfrp5 mRNA expression and protein secretion were increased during the differentiation of 3T3-L1 pre-adipocytes Upregulation of Sfrp5 expression and secretion in adipocytes is one crucial mechanism by which rosiglitazone and metformin improve IR	[134]
	Epicardial adipose tissue (EAT) and subcutaneous adipose tissue (SAT)	–	Sfrp5 mRNA levels were higher in EAT samples than in the paired SAT samples in both CAD and non-CAD group Sfrp5 is secreted by visceral fat and that its local concentration in EAT may greatly exceed that in SAT Low Sfrp5 and high Wnt5a levels are associated with the presence of CAD	[135]
	Rat	–	Sfrp5 overexpression reverses the effects of microRNA-199a inhibitor on proliferation, migration, and cardiac fibroblast-to-myo fibroblast transformation of cardiac fibroblasts	[132]
	Mice	–	Sfrp5 decreases the infarct size. Suppress pro-inflammatory Wnt5a/JNK signaling within the macrophages that infiltrate the infarct and pro-apoptotic Wnt5a/JNK signaling within myocytes	[123]
	INS-1E cells	–	Sfrp5 reduces markers of cell proliferation, increases parallelly dose-dependently glucose-stimulated insulin secretion in INS-1E cells	[134]
CTRPs	Wistar Rats	–	CTRP3 protein expression levels are decreased in VAT at the pathogenic stages of insulin resistance and in T2DM	[135]
	3T3-L1 adipocytes	–	CTRP12 improves the glucose metabolism 3T3-L1 adipocytes	[136]
	C57BL/6 mice	–	CTRP12 have anti-diabetic actions that preferentially acts on adipose tissue and liver to control whole body glucose metabolism	[137]
	CTRP1 transgenic (TG) mice	–	CTRP1 stimulated glucose uptake through the glucose transporter. GLUT4 translocation to the plasma membrane and also increased glucose consumption by stimulating glycolysis	[114]
	Rats	Jugular vein injection	CTRP9 attenuates atrial inflammation and fibrosis via toll-like receptor 4/NF-κβ and Smad2/3 signaling pathways	[138]
	Sprague–Dawley rats	Tail vein injection	CTRP3 protects cardiomyopathy via activating AMPKα pathway	[139]

**Table 2:** Clinical evidence of anti-inflammatory adipokines in insulin resistance and atherogenic dyslipidemia

Adipokines	Mode of Evaluation	Action	References
Adiponectin	Standard laboratory assessment of adiponectin, ESAM, ICAM1, and VEGF	Adiponectin serve as markers of endothelial dysfunction and neo angiogenesis	[140]
	Fasting total and HMW adiponectin were measured in 86 subjects from the Coronary Artery Calcification in T1D (CACTI) cohort	Adiponectin levels are positively correlated with insulin sensitivity in T1D patients Insulin sensitivity is lower for patients with T1D	[141]
	Plasma levels of adiponectin, the metabolic syndrome and the occurrence of small dense LDL particles	Decreased adiponectin levels is associated with increased small LDL particles	[142]
	25 non-obese individuals with low or normal IRS-1 expression in subcutaneous abdominal fat cells were extensively characterized and the results compared with 71 carefully matched subjects with or without a known genetic predisposition for type 2 diabetes	Subjects with low IRS-1 with insulin resistant shows increased carotid artery bulb intima media thickness vs those with normal IRS-1 protein expression	[143]
	Determination and correlate among adiponectin, IR and atherosclerosis in non-diabetic hypertensive patients and healthy volunteers	Low adiponectin levels positively correlate with decreased insulin sensitivity increased pro-inflammatory cytokine production and worsening atherosclerosis in hypertensive patients and healthy adults	[144]
	Determination of the correlation between plasma adiponectin concentration with insulin resistance and atherosclerosis	Adiponectin directly or indirectly improves insulin resistance Significant negative correlations are exist between adiponectin concentration with insulin resistance and atherosclerosis	[145]
	Adipocytokines, inflammatory biomarkers, parameters of insulin resistance, and lipid sub fractions determination in the early stages of atherosclerosis in juvenile	Serum adiponectin levels provide the evidence of early atherosclerosis linked to hypoadiponectinemia Adiponectin plays important role in the development of atherosclerosis	[146]
	Determination of circulation adiponectin levels, risk factors for atherosclerosis for the human volunteer with type 2 diabetes	Circulating levels of adiponectin were decreased in non-obese volunteer but with insulin resistance Hypoadiponectinemia plays an important link between cardiovascular disease and IRS	[147]
	48 men (aged 40–60) with angiographically confirmed coronary atherosclerosis and 19 healthy men, matched by age, as a control group were taken as sample	Lower adiponectin level is connected with resistance syndrome and atherogenic lipid profile	[148]
	Plasma adiponectin of diabetic patients and non-diabetic patients were compared	Higher levels of adiponectin are associated with lower cases of diabetic patients compared to diabetic patients	[149]
Omentin-1	Impact of omentin-1 in obesity induced diabetes mellitus	Omentin-1 level is decreased in obesity and diabetic condition Omentin-1 serve as important markers for the obesity and its associated comorbidities	[150]
	Patients with impaired glucose regulation, patients with untreated type 2 diabetes mellitus (T2DM), and subjects with normal glucose tolerance were enrolled in this study Serum omentin-1 and plasma glucose at fasting and at 2 h after glucose load and fasting serum levels of TNF-a, IL-6, insulin, and HbA1c were measured and compared	Decreased serum omentin-1 levels were observed impaired glucose regulation subjects Decreased levels of omentin-1 or lack of omentin-1 contributes to the development of insulin resistance and diabetes mellitus	[151]
	100 and 55 patients with CAD were divided into two groups: acute coronary syndrome (ACS) and stable angina pectoris (SAP). A total of 52 healthy participants served as controls The association of omentin-1 with CAD and cardiovascular disease risk factors was evaluated	Serum omentin-1 level is negatively associated with CAD	[152]
	The impact of 12 weeks of aerobic (cycle ergometer), resistance, and combined exercises on omentin-1 level, glucose and insulin resistance indices in overweight middle age women with T2DM	12 weeks of aerobic and resistance exercises improve HOMA-IR and increase serum omentin-1 among women with T2DM	[155]
	Omentin-1 with carotid intima-media thickness and metabolic markers were studied	Lower levels of Omentin-1 is closely associated with metabolic syndrome and play important role in the development of atherosclerosis in metabolic syndrome patients	[156]
	80 newly diagnosed female type 2 diabetic patients and 40 age matched female control subjects and comparison of plasma omentin-1 levels	Omentin-1 levels are low in type 2 diabetics and insulin resistant females Omentin-1 has very important link with metabolic disturbances such as obesity, insulin resistance and the regulation of omentin-1 in diabetic patients	[157]
	60 obese type 2 diabetic females and 30 healthy female subjects formed the control group were enrolled Fasting (blood glucose, insulin, lipid profile, omentin-1) and HbA1c were measured	Lower omentin-1 level was observed in patients with diabetes mellitus Serum omentin-1 can be used as a biomarker for obesity related metabolic disorders	[158]
	75 patients with 2 diabetes and 15 healthy control subjects were enrolled in this study Insulin levels, interleukin‐6, omentin‐1 and chemerin were compared	Omentin-1 and chemerin play important role in obesity and its associated disorders such as type 2 diabetes and cardiovascular disease	[159]
Sfrp5	Cross-sectional studies of Chinese population including 194 control participants and 90 metabolic syndrome patients	Sfrp5 is linked to metabolic syndrome	[160]
	Serum concentrations of Sfrp5, Wnt5a and adiponectin were measured in 47 individuals who participated in a coffee intervention study	Sfrp5 is directly related to HOMA‐IR and oxidative stress in humans	[161]
	185 patients suspecting CAD were included in the study and divided into two groups CAD and non-CAD groups as per their results of coronary angiography Serum Sfrp5 levels of the subjects were measured by ELISA	The serum sfrp5 levels in CAD were significantly lower than non-CAD patients The serum level of Sfrp5 was negatively correlated with body mass index, insulin resistance, and the severity of CAD	[162]
	104 healthy subjects, 101 with impaired glucose tolerance, and 112 with newly diagnosed type 2 diabetes mellitus and, in a separate study, 30 healthy women and 32 women with polycystic ovarian syndrome (PCOS) were included for the study. Oral glucose tolerance test and euglycemic-hyperinsulinemia clamp were performed to assess glucose tolerance and insulin sensitivity	Circulating Sfrp5 was significantly lower in both impaired glucose intolerance and newly diagnosed type 2 diabetes mellitus than in individuals with normal glucose tolerance	[163]
	58 type 2 diabetes patients, 22 latent autoimmune diabetes (LADA) in adults patients and 40 healthy controls were enrolled into this study ELISA was employed to detect the circulating Sfrp5 level in plasma, and other lab tests such as fasting glucose and creatinine were also examined	Circulating Sfrp5 level was significantly decreased in T2D and LADA patients plasma compared with that in healthy control Sfrp5 was correlated with homeostasis model assessment of insulin resistance (HOMA-IR), diabetes duration and BMI Sfrp5 was still negatively correlated with HOMA-IR after being adjusted for disease duration and BMI	[164]
	82 patients with T2DM and 42 non-diabetic subjects were enrolled for the study Plasma Sfrp5 and Wnt5a concentrations were measured through ELISA	Elevated Sfrp5 levels in uncomplicated type 2 diabetic subjects indicate that Sfrp5 may play a role in the pathogenesis of T2DM	[165]
	70 drug‐naïve T2D patients, 70 pre-diabetic subjects and 70 controls were enrolled for the study All subjects body mass index matched to the T2D patients and overweight or obese. Sfrp5, hormones and cytokines levels were measured by ELISA	Serum Sfrp5 levels were elevated in T2D patients as compared with pre-diabetic subjects No differences were found in serum Sfrp5 levels between pre-diabetic subjects and controls Circulating Sfrp5 levels were independently associated with T2D as compared with prediabetes and normal glucose tolerance state	[166]
	Two hundred eighty four subjects 90 with metabolic syndrome and 194 healthy controls, 153 men and 131 women	Circulating levels of Sfrp5 was significantly lower in newly diagnosed metabolic syndrome patients than in control subjects Sfrp5 may be an adipokine which is associated with the pathogenesis of metabolic syndrome in humans	[159]
CTRPS (CTRP1, CTRP3, CTRP9, CTRP13, etc.)	Serum CTRP3 levels, anthropometric, inflammatory and metabolic parameters were measured in 180 obesity and essential hypertensive patients and in 66 normal weight, normotensive subjects	The serum CTRP3 levels in the obesity group were lower than those in the normal weight group These levels were also lower in hypertensive subjects than in normotensive subjects CTRP3 was an independent factor affecting blood pressure and IR and may play an important role in the pathogenesis of obesity and hypertension	[167]
	135 subjects were recruited to this study, including 62 type 2 diabetic patients (DM group) and 73 healthy subjects (control group) Biochemical parameters, CTRP1, TNF-α and adiponectin were measured using enzyme-linked immunosorbent assay (ELISA)	Plasma CTRP1 levels difference were observed between the DM group and the control group CTRP1 was strongly positively associated with BMI, glucose levels, HbA1c, HOMA-IR and TNF-α in diabetic patient CTRP1 share similar actions of adiponectin but exhibit opposite compensatory upregulation in the diabetic state	[168]
	Plasma CTRP1 level was investigated in type 2 diabetic subjects (35) and non-diabetic subjects (35) The relationship between CTRP1 and phosphorylation of multi insulin receptor substrate 1 (IRS-1) serine (Ser) sites was further explored	Plasma CTRP1 was higher and have negative correlation with insulin resistance in diabetic subjects Glucose utilization test revealed that the glucose utilization rate of mature adipocytes was improved by CTRP1 in the presence of insulin	[169]
	CTRP1 serum levels in 539 patients undergoing *coronary angiography* for the evaluation of established or suspected stable CAD	CTRP1 is associated with obesity-linked disorders CTRP1 is associated with major adverse cardiovascular events CTRP1 is associated with cardiovascular risk beyond its association with obesity-linked disorders	[170]
	Study participants were divided into two groups according to the results of coronary angiography: a control group (63) and a CAD group (76). The concentrations of serum CTRP1 and inflammatory cytokines were determined by ELISA	Serum levels of CTRP1 were significantly higher in CAD patients than in controls, and CTRP1 levels increased with increasing severity of CAD CTRP1 levels with the prevalence and severity of CAD, indicating that CTRP1 can be regarded as a novel and valuable biomarker for CAD	[171]
	357 consecutive patients who had stable angina and at least one lesion with 100% occlusion between January 2010 and September 2012 were screened Blood samples were collected on the day of angiography after overnight fasting. Serum levels of CTRP1, CTRP3 and high-sensitivity C-reactive protein (hsCRP) were assayed using ELISA kits	Association between increased serum CTRP1 level and low coronary collateralization in patients with stable angina and chronic total occlusion were observed. CTRP1 inhibits in vitro angiogenesis of endothelial progenitor cells from patients with severe coronary artery disease	[172]
	Serum CTRP3 levels, anthropometric, inflammatory and metabolic parameters were measured in 180 obesity and essential hypertensive patients and in 66 normal weight, normotensive subjects	The serum CTRP3 levels in the obesity group were lower than those in the normal weight group; these levels were also lower in hypertensive subjects than in normotensive subjects CTRP3 was an independent factor affecting blood pressure and IR, and may play an important role in the pathogenesis of obesity and hypertension	[167]
	Cross-sectional study performed on 55 controls, 54 patients with T2DM, and 55 patients with T2DM-normal patients. Serum levels of CTRP3, adiponectin, TNF-α, and IL-6 were measured by ELISA technique	Serum levels of CTRP3 were significantly lower in patients with T2DM and T2DM-normal patients Decreased serum levels of CTRP3 in patients with T2DM and diabetic nephropathy and its association with pathologic mechanisms in these patients suggested a possible role for CTRP3 in pathogenesis of diabetic nephropathy	[173]
	Circulating progranulin and CTRP3 concentrations in 127 subjects with (44) or without metabolic syndrome (83) The relationship of progranulin and CTRP3 levels with inflammatory markers and cardiometabolic risk factors, including high-sensitivity C-reactive protein (hsCRP), interleukin-6 (IL-6), estimated glomerular filtration rate (eGFR), and adiponectin serum concentrations, as well as carotid intima-media thickness, was analyzed	Circulating progranulin levels are significantly related with inflammatory markers, hsCRP, whereas CTRP3 concentrations exhibit a significant association with cardiometabolic risk factors	[174]
	Subjects with normal glucose tolerance, impaired glucose tolerance and newly diagnosed type 2 diabetes mellitus were recruited to determining the circulating CTRP9 and adiponectin levels by ELISA	Circulating CTRP9 level was higher in both impaired *glucose* *intolerance* and newly diagnosed *T2DM* than in individuals with normal *glucose tolerance*. Overweight subjects had higher CTRP9 levels than lean individuals, and in all subjects, females also had higher CTRP9 levels than males. Circulating CTRP9 level was positively correlated with markers of obesity and insulin resistance	[175]
	337 subjects who underwent coronary angiography and were categorized into four groups according to the presence of CAD and T2DM (control, CAD, T2DM and CAD + T2DM) Serum levels of CTRP9, adiponectin, sICAM-1, sVCAM-1, sE-Selectin, IL-6 and TNF-α were measured	The circulating CTRP9 levels were independently associated with increased risk of CAD and T2DM in addition to elevated levels of serum CTRP9 in CAD, T2DM and CAD T2DM groups Circulating levels of CTRP9 in T2DM and CAD individuals which suggests a compensatory response to insulin resistance, inflammatory milieu and endothelial dysfunction	[176]
	Circulating levels of CTRP13 and adiponectin were measured by \ELISA in T2D patients (40) and in an age and gender-matched control group (*n* = 40)	Circulating levels of CTRP13 and adiponectin were significantly lower in T2D patients in comparison with controls CTRP13 is a novel adipokine associated with T2D in humans as its serum level was significantly lower in T2D patients and was inversely correlated with insulin resistance	[177]
	Plasma levels of CTRP13 in healthy control and patients with NAFLD, T2DM and NAFLD + T2DM, and also correlations between CTRP13 plasma levels and clinical and subclinical features Circulating CTRP13 was examined in 88 male (20 healthy controls, 22 T2DM patients, 22 NAFLD patients and 22 NAFLD + T2DM patients). CTRP13 and adiponectin plasma levels were measured by ELISA method	CTRP13 serum levels were higher in the control group than the other groups CTRP13 had significant negative correlation with unfavorable anthropometric and metabolic factors including BMI, visceral fat, Insulin, HOMA-IR, TG, AST, ALT and ɣ-GT and have a positive correlation with plasma concentration of adiponectin	[178]
	Serum levels of CTRP3, CTRP13, adiponectin and inflammatory cytokines and their gene expression in peripheral blood mononuclear cells (PBMCs) were determined in 172 subjects categorized as group I (without T2DM and CAD), group II (with CAD but no T2DM), group III (with T2DM but no CAD) and group IV (with T2DM and CAD)	Serum levels and gene expression of CTRP3, CTRP13 and adiponectin in the group I were higher compared to other groups This suggests emerging role of these adipokines in the pathogenesis of CAD	[179]

It is important to know that the former effects are exerted by AAKs whereas later by PAKs, whereas many adipokines function are yet to be reported. Most of the adipokines are derived from either VAT and subcutaneous adipose tissue (SAT) [51, 105]. Although there are numbers of AAKs and PAKs that act directly and indirectly on metabolic health of humans, in this article the adipokines which are actively and mostly found to be associated with atherogenic dyslipidemia and insulin resistance are considered for discussion. The PAKs are upregulated during obesity and can promote obesity-linked CMDs. Most of the PAKs that researchers think to be involved with the metabolic diseases are leptin, TNFα, IL-6 and resistin. Alternatively there are AAKs that are thought to be useful in the prevention or therapeutic intervention of the metabolic diseases are adiponectin, omentin-1, some members of CTRP family and Sfrp5. The level of these PAKs and AAKs changes in metabolic complications; therefore, function and therapeutic intervention of the adipokines/or with the adipokines can be a game changer in the management or therapeutic prospects and their potential utility as a biological marker in the management of CMDs.

### Pro-inflammatory adipokines (PAKs)

#### Tumor necrosis factor (TNF-α)

TNF-α is secreted from myeloid cells via activation of mitogen-activated protein kinase (MAPK) and NFkB signaling and responsible for secretion of other inflammatory cytokines, e.g., IL-1 and IL-6 [106]. It is the first WAT-derived PAKs reported to involve in initiation and progression of insulin resistance [26]. TNF-α are released by AT-resident macrophages and found to be overexpressed in obese animals AT [107]. It was observed that mice lacking TNF-α or its receptor are resistant to the development of insulin resistance [108]. TNF-α is higher in AT in obese human subject and positively correlated with insulin resistance[109, 110]. Long term treatment of anti-TNF-α inhibitor treatment patients with metabolic syndrome reported to be improved in fasting blood sugar and increased adiponectin levels[111]. TNF-α is involved in phosphorylation of IRS-1 receptors and has direct negative inference in the insulin signaling pathway [112]. TNF-α also affects the adipocyte differentiation and lipid metabolism, thereby indirectly induces insulin resistance. TNF-α increases hepatic glucose production due to its action in promoting lipid metabolism and secretion of free FA [113]. TNF-α hinders the conversion of pre-adipocyte to mature adipocytes through the downregulation of adipogenic genes such as peroxisome proliferator-activated receptor gamma (PPAR-γ) and CCAAT/enhancer binding protein (C/EBP) thus leads to expansion of AT mass [114]. TNF-α also activates NF-κβ genes and downregulates mRNA levels of adiponectin [115, 116]. However, the effect on immune response of TNF-α is mainly due to the enhancing secretion of other cytokines, such as IL-6, rather than direct effect [117].

#### Leptin

Leptin is 16-kd protein and was identified in obese gene (ob)of ob/ob mice [118]. Leptin is AT specific adipokines that regulates appetite, energy expenditure, behavior and glucose metabolism [119]. Mice lack of leptin shows hyperphagia, obesity, and insulin resistance. However, delivery of leptin in ob/ob mice reverses the conditions [120]. When leptin is injected to ob/ob mice, it has multiple beneficial effects in health such as reduction in food intake, body mass, increased it has shown rapid reduction in food intake, body mass, increased energy expenditure and restored euglycemia [121]. However, leptin is positively correlated with AT mass, obesity and increased levels of leptin does not have any expected decrease in food intake, signifying that leptin resistance occurs during obesity [120]. In normal circumstances, leptin mediates its anorexic actions in hypothalamus, by binding to the leptin receptor b (LRb) and through the activation of janus kinase 2/ Signal transducer and activator of transcription 3 (JAK2/STAT3) signaling. However, in obesity this pathway is blocked by several mechanisms. One of the mechanisms includes, STAT3-mediated induction of SOCS3 protein, impairs leptin induced signaling by binding to phosphorylated Tyr985 residues of LRb [122]. Animal studies proved that SOCS3 is responsible for leptin resistance [123]. In inflammation leptin levels are increased in AT as well as in serum and acts on monocytes/macrophages, neutrophils, and T cells, and enhance the production of the pro-inflammatory cytokines and suppresses anti-inflammatory cytokines [124, 125]. Leptin suppresses the production of TH-2 type cytokine, IL-4 and increases the TH1 type cytokines and polarized T cells towards TH1 phenotype [124, 126]. Many preclinical and clinical studies have proved the link of leptin with atherogenesis and metabolic syndrome. Circulating levels of leptin is positively correlated with metabolic syndrome and cardiovascular disease [127]. Increased leptin levels significantly alarms the pathogenic risk of coronary heart disease (CHD) [128]. Leptin levels are increased after myocardial infarction in humans [129]. Greater cardiac hypertrophy was observed in leptin deficient mice and provided greater cardiac remodeling in response to chronic ischemic injury [130, 131].

Leptin shows both insulin sensitizing and insulin resistance effects. However, these effects if we consider directly attributed to leptin is debatable. This is because of AT, a dynamic endocrine organ where when leptin concentration changes, may lead to changes in other metabolically active hormones also [132]. Leptin acts both peripherally (skeletal muscle, liver, pancreas, and fat) as well as centrally via central nervous system (CNS) to control basal and insulin-mediated glucose homeostasis. *In-vitro* studies suggest that leptin has an important inhibitory role in glucose metabolism. However, insulin sensitizing effect also has been proposed in *in-vivo* studies which depends on the central mechanism.

#### Interleukin-6 (IL-6)

IL-6 is a versatile, pleiotropic adipokine reported to be engaged in vital roles such as regulation of inflammation, hematopoiesis, immune responses, and host defense mechanisms [133]. It is a PAK, and AT is responsible for secretion of 15–30% of IL-6 in normal healthy people [134].IL-6 is produced by macrophages, fibroblast and the stromal vascular fraction of visceral WAT [51]. VAT releases more IL-6 than SAT and acts as a marker for visceral adiposity [120]. IL-6 is one of the major PAK which is actively involved in chronic inflammatory disease such as atherosclerosis [135]. Genetic polymorphism studies have confirmed the linkage of IL-6 receptor signaling and its association with CAD [136]. IL-6 levels are positively correlated with increased risk of MI [137]. Further, IL-6 and its receptor are linked to plaque instability [138]. It is believed that production of IL-6 is stimulated by TNF-α.

The link between obesity and T2D has been well documented and suggests the relation between obesity and insulin resistance. It should be noted that circulating levels of IL-6 is two or three fold higher in obese patients with T2D compared to normal person [139]. However, obesity and its link to metabolic syndrome is controversial [140]. Some researchers suggest the existence of a relationship with elevated levels of IL-6 and insulin resistance or T2D [141, 142]; however, several argue against the existing relationship. They suggest that increased fat mass and elevated IL-6 levels are not independent risk factors for development of insulin resistance [143]. This is because visceral fat releases a much higher quantity of IL-6 and is a stronger predictor of diabetes than total fat mass [144].

#### Resistin

Resistin is 10 KDa polypeptide with 114 amino acids in roden, similar in molecular structure to adiponectin and first identified in obese mice, affects in glucose homeostasis and mediate insulin resistance [117, 145]. Large population based studies confirm the positive correlation between circulating resistin and fasting serum TG [146]. Resistin levels are increased in obesity and insulin resistance in rodents [147]. Insulin resistance is mainly due to the interference in normal insulin signaling by decreasing the expression of insulin receptors, IRS1 and IRS2 [148]. Resistin also decreases the activation of AMPK which is a potential insulin sensitizing molecule [149]. Recombinant resistin administration to normal animals produce insulin resistance, however, immune neutralization of resistin improves insulin sensitivity in obese animals with insulin resistance [147]. Resistin injures endothelium by inducting adhesion molecules VCAM-1 and MCP-1 expression and secretions and synthesizing endothelin-1 by endothelial cells [72]. Insulin resistance in humans by resistin is not clear as in rodents. Resistin is expressed in macrophage in humans, signifying a pro-inflammatory action rather than their involvement in glucose metabolism. Resistin induces oxidative stress and inhibits eNOS in human endothelial cells [150]. In human macrophages, resistin support foam cell formation and induce platelet activation by increasing P-selectin expression [151, 152]. Therefore, the findings suggest that human resistin might play an important role in development of atherosclerosis.

#### Visfatin

Visfatin is produced mainly by the adipocyte in visceral AT. It is a 52 kDa multifunctional protein with several activities. Visfatin, also known as nicotinamide phosphoribosyl transferase (NAMPT), or pre-B cell colony-enhancing factor (PBEF), is known to play a crucial role in regulating numerous pathophysiological functions [153]. In metabolic disease, circulating visfatin level increases and has been positively correlated with cardiovascular diseases. High plasma levels of visfatin are also associated with vascular inflammation, endothelial dysfunction and atherosclerotic plaque destabilization [154].

### Anti-inflammatory adipokines(AAKs)

Adipokines have diverse functions depending on their properties. However, there are certain adipokines that are beneficial for human health and categorized as AAKs. Numbers of adipokines are available with their categorized functional properties, but in this paper we are discussing those AAKs which have direct or indirect impact on the metabolic health considering atherogenic dyslipidemia and insulin resistance as reference. The reason for choosing few adipokines can be explained by their exploratory role mainly on atherogenesis, and insulin resistance.

#### Adiponectin

Adiponectin is adipocyte-derived hormones comprising of four distinct domains, e.g., a signal peptide at the N terminus, a short variable region, collagenous domain and a C-terminal globular domain homologous to C1q [155]. Mouse and human adiponectin have 83% homology and contain 247 and 244 amino acid sequences, respectively [156]. The crystal structure of adiponectin is similar to that of TNF-α [157]. Adiponectin and C1q/TNF-related protein (CTRP) share the common structure as mentioned earlier. Adiponectin exists in three multimeric forms: a trimer, low molecular weight (LMW), a hexamer medium multimer and larger multimeric high molecular weight (HMW) [156, 158]. Adiponectin is secreted by adipocytes and its expression is ≈100 fold during adipocyte differentiation [159]. In healthy adults, the adiponectin concentration varies in human serum from 1.9 to 17.0 g/ml [159]. Plasma level of adiponectin in healthy people or mice is 1000 times higher than leptin accounting 0.01% of total plasma protein [160]. Adiponectin is a well-established biomarker of increased risk of insulin resistance, CVDs, etc. [161]. Despite adiponectin being secreted exclusively by AT, during obesity the level of adiponectin decreases, but paradoxically increases during caloric restriction (CR), anorexia nervosa (AN). The paradox of adiponectin may be explained in this way that in insulin resistance or obesity with insulin resistance state, decreased adiponectin may results from the decreased expression and transcript protein of adiponectin which may be from mitochondrial dysfunction, hypoxia and or ER stress [162]. However, the increased expression of adiponectin in CR and AN remained unclear although few studies have shown increased expression of adiponectin in extensive CR [163]. Most of the study including animals and humans reported that serum adiponectin levels are increased with prolonged CR and weight loss, but not from the WAT or without affecting expression or secretion in WAT [164, 166].  Moreover, the human subject shows decrease in adiponectin expression in WAT during AN and clearances of adiponectin remain unaltered during CR [163, 166]. Moreover, changes of circulating adiponectin in response to treatment with insulin or thiazolidinedione are also not related to adiponectin transcript expression in WAT [167]. The question is during CR or AR, where does adiponectin come from if the expression of adiponectin remains unaltered in WAT? The question remained unanswered until Cawthorn et al. investigated the bone marrow AT (MAT) that secret adiponectin in the circulation [168]. In normal healthy subjects, MAT comprises 13% of total adipose mass, where as in AN, 31.5% MAT clearly suggest that the expansion of MAT. In AN subject, MAT comprises 30% of total body fat and is sufficient to be a major contributor of adiponectin to the circulating adiponectin [168]. Using Wnt10b mice with specific MAT ablation with CR, shows increased resistance in both MAT and serum adiponectin without having any impact on WAT mass as well as adiponectin expression in WAT. On the other hand, MAT expansion increases serum adiponectin and adapts skeletal muscle during CR. Thus, all the evidence gives conclusive results that MAT is a key source of adiponectin and reaches the circulation through endocrine action [168].

Adiponectin regulates endothelial function by influencing adhesion and transmigration of leukocyte and macrophages which are mediated by ICAM1, VCAM and E-selectins. Adiponectin level is decreased in obesity and in insulin resistance and low adiponectin levels are found to be associated with endothelial dysfunction [169]. Animal disease model and *in-vivo* study confirms the lower adiponectin level exacerbates vascular injury and overexpression of adiponectin protects from atherosclerosis [170, 171]. Adiponectin protects vascular endothelium by anti-inflammatory action against oxidative stress and inflammatory cytokines suggests molecular mechanism involves mainly inhibition of inflammatory signal *in-vivo *[172]. Adiponectin deficiency enhances leukocyte–endothelial cell interactions via reduced availability of eNO at the vascular wall and upregulation of endothelial CAMs, leading to vascular inflammation and atherosclerosis [61]. Administration of pharmacologically active doses of the recombinant globular adiponectin (gAd) reverts the endothelial dysfunction associated with adiponectin deficiency and attenuates cytokine-induced vascular inflammation in wild type (WT) mice and maintains the expressing of physiologic concentrations of adiponectin in the blood [61]. Adiponectin deficiency increases the leukocyte rolling and adhesion. Increased leukocyte rolling flux decreases the velocities of rolling leukocytes and increases the adhesion to the vascular wall. WT mice when treated with gAd, show normalized leukocyte rolling flux, leukocyte rolling velocity and leukocyte adhesion which supports the hypothesis that vascular inflammation due to adiponectin deficiency may be treatable with the with similar adiponectin isoforms, i.e., gAd [61]. gAd has been reported to reverse the TNF-α induced leukocyte-endothelium interactions in WT mice. TNF-α downregulate eNOs/NO signaling and upregulates endothelial CAM [66, 173]. Treatment with gAd inhibits TNF-α mediate leukocyte–endothelial interaction and reverses the TNF-α signaling in endothelial cell culture study [61, 174]. Endogenous adiponectin and gAd regulates the availability of NO in endothelium. Adiponectin deficiency shows 40% reduction in eNO availability, and treatment with gAd maintains the physiological levels of adiponectin. The ability to suppress TNF-α till 55% clearly demonstrates the anti-inflammatory action of adiponectin [61]. The ability to mitigate the anti-inflammatory effect in endothelium, suppression of CAM and availability of eNO reflects the possibilities of anti-atherogenic activity of adiponectin, thereby cardioprotection.

Adiponectin exerts its anti-inflammatory action through its receptor Adiponectin R1 (adipoR1), adiponectin (adipoR2) and T-cadherin [175]. Numbers of study reported direct action of adiponectin on inflammatory cells and NF-κβ. Adiponectin suppress foam cell transformation from macrophages by inhibiting the function of mature macrophages [176], stimulates the macrophage production of anti-inflammatory cytokine IL-10 and inhibits TNF-α induced VCAM-1, E-selectin expression on endothelial cells [177], inhibits NF-κβ activation in macrophages which is induced by TLR [178]. Adiponectins action on NF-κβ is complex presenting both inhibitory as well as stimulatory effects. Adiponectin possess inhibitory action on NF-κβ, inhibits lipopolysaccharide (LPS) induced NF-κβ activation in adipocytes [179],TNF-α induced NF-κβ pathways in endothelial cells [174]and NF-κβ pathway in macrophage [180]. Inhibition of NF-κβ pathway results in anti-inflammatory action of adiponectin and decreases the pro-inflammatory cytokines. On the other hand, the action of gAd and high molecular weight (HMW) adiponectin were compared on NF-κβ pathways in vascular endothelial cells [181]. High molecular weight (HMW) adiponectin when undergoing proteolytic cleavage forms globular adiponectin. HMW adiponectin activates NF-κβ modestly compared to gAd which activates very strongly. HMW requires a shorter period to inhibit TNF-α induced NF-κβ activation, whereas gAd induces expression of various PAKs, adhesion molecules and requires a longer period to inhibit cytokine-induced NF-κβ activation. Therefore, HMW adiponectin may act as an anti-inflammatory whereas cleavage of adiponectin at an inflammatory site may enhance inflammation. However, the dual nature of adiponectin is not clearly understood, and questions remain unresolved regarding the timing of the effects.

Researchers have unveiled the link between adiponectin and its microvascular connection in the regulation of insulin. Skeletal muscle acts as a major organ participating in insulin stimulated glucose metabolism accounting 80% of total body glucose [182]. Insulin is secreted by the pancreatic β-cells, and to act in the muscle it has to be delivered to the muscle cells via capillaries nurturing the muscle cells followed by transportation through the capillary endothelium which enters interstitial space where they bind to the insulin receptor called myocyte to exert metabolic action [183].

Muscle microvasculature plays critical roles in the regulation of insulin secretion in muscle. Insulin action in the muscle cells starts, when it is delivered to the capillaries which nurture the muscle cells, followed by transportation of insulin through capillaries of endothelium to enter the interstitial space [184]. Microcirculation comprises all vessels including venules, arterioles and venules (< 150 µm in diameter). Their functions are to deliver and exchange an adequate amount of nutrients, hormones, oxygen, between the plasma and tissue interstitium. During normal or rested state approximately 30% of the capillaries are functionally perfused, but in response to increased demand especially during exercise more capillaries become functionally perfused via more relaxation of the pre-capillary terminal arterioles [82]. This process is called microvascular recruitment. Insulin mediated microvascular recruitment dispossesses insulin mediated glucose in muscle and blocks the insulin's action on microvascular recruitment. It is reported that insulin-mediated capillary recruitment in skeletal muscles is impaired with diabetes mellitus (DM) [185]. A clinical study reported that obesity blunts the insulin mediated microvascular recruitment in forearm muscle. They assumed that the blunted recruitment in obese individuals are involved at least one part of the insulin mediated glucose disposal and absence of microvascular response [186]. Therefore, insulin and microvascular are appeared to be important for enhancing delivery of insulin and glucose to skeletal muscle and the impaired responses to insulin in the obese subjects might contributes impaired metabolic response. Adiponectin is a potent vasodilator and the action is mediated via NO-dependent mechanisms [187]. Adiponectin modulates muscle insulin action and the expansion of endothelial exchange surface area due to its potent vasodilatory effect via NO-dependent mechanism [183, 187]**.** Muscle microvasculature is the regulatory site of insulin’s metabolic action and mounting evidence suggests that since adiponectin has both vasodilatory and insulin sensitizing actions, adiponectin modulate microvascular recruitment thereby insulin delivery as well as action in muscle [183].

#### Omentin-1

The endemic problem of the T2DM is a major problem associated with the modern sedentary lifestyle. Importantly, early diagnostic tools are needed for detection of insulin resistance. Moreover, novel therapeutic agents also need to be explored. One such molecule is omentin-1. It has multiple activities including insulin-sensitizing activity. Omentin-1 is a novel 34KDa adipokine first identified in human omental AT, also called intestinal lactoferrin receptor [188, 189]. The physiological, pathophysiological and clinical features of omentin-1 have gained attention due to its experimental and clinical evidence showing its involvement in metabolic disorders [190, 191]. In obesity, plasma omentin-1 and mRNA expression was decreased in VAT [192]. Reduced omentin-1 levels are found to be closely related to metabolic syndrome in morbidly obese women [193]. The expression of omentin-1 is most abundantly found in epicardial adipose tissue (EAT) and visceral fat surrounding the heart and coronary arteries [194]. EAT is attached to the myocardium. Therefore, omentin-1 secreted in EAT directly affects the cardiac function [195]. Omentin-1 suppresses ICAM-1, VCAM-1 and cyclooxygenase-2(COX-2) in human umbilical vein endothelial cells (HUVECs) through ERK/NF-kβ, JNK/AMP-activated protein kinase (AMPK), and eNOS signaling pathways [196, 197]. Omentin-1 does not affect monocyte differentiation to macrophages but is responsible for shifting the balance differentiation preferentially in favor of anti-inflammatory M2 macrophages instead of M1 phenotype [198]. Omentin-1 level is negatively correlated with waist circumference, BMI, systolic blood pressure, carotid intima-media thickness, stiffness, and insulin resistance [199]. It inhibits vascular inflammation and pathological remodeling that are involved in the development of atherosclerosis and also possesses vasodilatory effects as well. Omentin-1 suppresses oxidation of LDL thereby inhibiting the formation of foam cell by downregulating scavenger receptors like CD36, scavenger receptor type A and the ratio of acyl-coenzyme A and cholesterol acyl-transferase-1 in human monocyte-derived macrophages [198].

It is well documented that omentin is a protective adipokine for CVD as it induces vasodilation, reduces endothelial dysfunction, and inhibits vascular inflammation and angiogenesis. These beneficial effects of novel adipokine omentin can be expected to play more roles in the protection of CVD in the future.

#### Secreted frizzled-related protein 5 (Sfrp5)

Secreted frizzled-related protein 5 (Sfrp5) is an adipocytokine, highly expressed in mature adipocytes of WAT [200] and its detectable in plasma [201]. It inhibits wingless-type family member 5A (WNT5A) signaling pathways, including non-canonical WNT5A/Ca2 + and WNT5A/c-jun N-terminal kinase (JNK) signaling pathways [202]. The expression of WNT5A has been reported to play a crucial role in the development of obesity, T2DM and atherosclerosis [203]. The link between obesity, insulin resistance and T2DM has been discussed in many research articles. Insulin resistance is considered as the main responsible factor involved in the pathogenesis of T2DM. Insulin resistance is a low grade inflammation linked to macrophages mediated inflammation in AT [26]. Sfrp5 is an anti-inflammatory adipokine which is capable of inhibit endogenous WNT5A pathways, might be effective to prevent macrophage mediated inflammation in AT to improve insulin sensitivity, thereby prevent development of T2DM [204]. Mice lacking Sfrp5 show impaired glucose clearance with high macrophage mediated AT inflammation and reduced insulin sensitivity, however, administration of Sfrp5 increases insulin sensitivity [200]. Furthermore, upregulation of Sfrp5 in 3T3 –L1 adipocyte cell line prevents inflammation and insulin resistance via blocking WNT5A. Although preclinical study in animal and cell line shows the protective role of Sfrp5 in T2DM, but clinical study has shown controversial results. Therefore, it is necessary that Sfrp5 deserves more clinical study with a large sample size, along with many ethnic group to further explore its role.

The involvement of Sfrp5, in cardiometabolic health, deserves more exploration. Serum levels of Sfrp5 are decreased in patients with CAD indicating the association of the adipokines in atherosclerosis [204]**.** Depletion of Sfrp5 in mice causes cardiac ischemia reperfusion injury along with increased inflammation and higher rates of cardiomyocyte deaths. Deficiency of Sfrp5 enhances WNT5A influx into the ischemic limb and also impairs revascularization [205]. Numbers of studies have demonstrated the atheroprotective role. Low serum levels of Sfrp5 are linked to CAD [206]. Sfrp5 were found to be inversely associated with multiple CMDs [207]. Higher levels of Sfrp5 inhibit endothelial dysfunction and arterial stiffness via downregulating Wnt5a/JNK pathways with reduced NO production [208]. The evidence provided by the different studies suggests that Sfrp5 may attenuate cardiometabolic symptoms and can be useful in the treatment or management of cardiometabolic diseases.

#### C1q/TNF-related proteins (CTRPs)

CTRPs are a new family of secreted proteins which have sequence homology with the adiponectin [208]. Till now 15 functional CTRPs have been identified which have different actions [209]. Out of 15, only a few numbers of CTRP have been ascribed to have implication in metabolic disorders whereas many others are still under investigation. All the CTRPs have common feature with four distinct domain, namely a signal peptide at N-terminus sequence, a short non-homologous or variable region, a collagenous domain consist of variable numbers of Gly-X–Y repeats and C-terminal globular domain homologous to complement factor C1q domain [210]. Most CTRPs are expressed in AT and can be detected in plasma. CTRPs have unique biological and signaling properties and they exist in the circulation as trimmers, assembling themselves into hexamaric and high molecular weight oligomeric complexes with their basic structural unit [211].

Sex, age and genetic background modulate the metabolic hormone levels as well as signaling pathways in both human and animals, and thus have variable impact in the development obesity and other metabolic disorders such as insulin resistance, and T2D [212, 213]. Interestingly, most of the CTRPs also circulate in the blood with variable concentration as per the sex and genetic background. A study reported that serum levels of adiponectin, CTRP1, CTRP2, CTRP3, CTRP5 and CTRP6 in six different genetic background mice showed significant variation [214]. The selected strain for the study was taken with varying degrees of susceptibility to insulin resistance or diabetes or diet-induced obesity. Biological activity of CTRPs depends on their multimeric forms. All CTRPs exist as trimer forms, however, accumulating evidence suggests that CTRPs, e.g., CTRP3, CTRP5, CTRP9, CTRP6, CTRP8, CTRP10, CTRP11, CTRP12, CTRP13 and CTRP15 happen to occur into multimeric complexes, via N-terminal cysteine residue or by oxido-reductase [207]. Adiponectin and CTRP 9 assemble to heterotrimers and exert the same biological action, i.e., cardioprotection via the same receptor [214]. Apart from forming as homo-oligomer, CTRP6/ CTRP1, CTRP7/CTRP2, and CTRP2/adiponectin form heterotrimers and generates functionally distinct ligands for secreted glycoproteins to provide new outline of action in normal and disease condition [215]. CTRP 9 exists as two isoforms namely 9A and 9B and CTRP 9B requires interaction with CTRP 9A and adiponectin for its action [216].

CTRPs are secreted as hormones and subjected to post translational modifications at their highly conserved residues. CTRP 12 has isomeric forms after post translational modifications such as glycosylated on the 39^th^ asparagine amino acid and 85^th^ cysteine modified with oligosaccharides [217]. The two isomeric forms of CTRP12 diverge from the oligomeric structure and function. It is reported that full length CTRP 12 activates Akt signaling in adipocytes, however, the globular form activates the MAPK signaling [218]. Adiponectin exists in multimeric forms where trimers and hexamers activate AMPK signaling in muscle thereby enhancing glucose uptake, deposition of glycogen as well as fatty acid oxidation. However, high molecular weight oligomers act on the liver and decrease glucose production [219]. Distinctively, CTRP1 and CTRP 2 are primarily secreted as trimmers in transfected HEK-293 cells. Primarily, CTRP2 in the mouse serum was found to be trimer form. Though CTRP3 secreted as trimmers, hexamers and HMW oligomers in transfected cells, it exists as HMW oligomers in mouse serum. Similar to CTRP3, CTRP5 also secreted in their multimeric forms but exists as trimmers in mouse serum. During exercise and treatment of metabolic complications such as obesity, T2DM, etc., the ratio of oligomeric CTRPs changes. The ratio of HMW and trimers CTRPs has been reported to serve as an index of insulin sensitivity. However, it is still required to determine whether metabolic disorders hinder the distribution of CTRPs oligomeric forms presence in the serum and their biological activities of these oligomeric proteins [220].

#### CTRPs reported to possess biological activity

Out of several CTRPs many of them possess biological activities and may be beneficial in the management or treatment of dyslipidemia and insulin resistance. CTRP1 has important roles in glucose metabolism by activating serine/threonine protein kinase Akt and MAPK p42/44 signaling in mouse myotube [210]. CTRP1 has been reported to possess anti-thrombotic properties and blocks platelet activation and aggregation by specifically binding to fibrillar. CTRP1 shows anti-thrombotic action by indirectly acting on the von Willebrand factor. CTRP1 creates an environment where less binding efficient COL-III is formed by inhibiting binding of the A3 domain of von Willebrand factor to COL-I without affecting the association of the A3 domain with platelet [214]. Therefore, the anti-thrombotic activity of CTRP1 may protect MI and stroke following rupturing of atherosclerotic plaques [214]. CTRP1 has been reported to prevent neointimal formation following arterial injury via a cAMP-dependent pathway by suppressing vascular smooth muscle cell growth [221]. In obesity and hypertension, inflammatory cytokines induce CTRP1 where there is a deficiency of adiponectin. Drug rosiglitazone found to be elevating CTRP1 level. Since CTRP1 administration reduces the blood glucose; it can be considered that the increased CTRP1 in obesity may be the compensatory action towards its resistance [205]**.** The pre-clinical and clinical data of CTRPs family members are been listed in Tables 1 and 2.

## Conclusions

As obesity is responsible for various diseases, including CVDs and metabolic disorders. Management of obesity and its co-morbid diseases are major challenges for the medical community. Alteration of the normal physiology of microcirculation in AT builds favorable conditions for the development of CMD. The knowledge of AT microcirculation is necessary to understand the underlying mechanism that regulates metabolic health. Despite the advancement of anti-obesity drugs, the main objective of sustained and non-recurrent weight loss could not be achieved due to the variable efficacy. Inherent side effects of drugs and poor patient compliance is also a major issue.

We are still in quest of an ideal agent for the management of obesity to prevent its comorbidities. Adipokines represent a very promising avenue in this regard. AAKs have a profound protective effect against metabolic risk. These agents conserve the normal physiology in AT microcirculation, prevent hypoxia and block polarization of M1 macrophage. AAKs suppress the oxidative stress and reduce ER stress via numerous pathophysiological pathways. AAKs are very potent anti-obesity molecules, higher levels of AAK in leaner patients in comparison to obese patients, and patients with disturbed lipidemic profile substantiate their anti-obesity and anti-atherogenic potential. Although the clinical efficacy of the AAKs is under the pipeline of research and development, some of the promising adipokines that can act as promising therapeutic agents include adiponectin, omentin-1, Sfrp5 and a few members of CTRP family which are shown in Tables 1 and 2.

Adiponectin is beneficial agents for obesity, as they inhibit gluconeogenesis in hepatocytes, thus controlling the deposition of fat. It also modulates angiogenesis and endothelial function and plays a crucial role in metabolic disorders like insulin resistance through the AMPK pathway. It also has an anti-atherogenic and anti-thrombotic effect, and thus if used for therapeutic purposes, it can be beneficial for management and treatment of metabolic disorders.

Similarly, omentin-1 is also a novel adipokine. It suppresses ICAM-1, VCAM-1, COX-2 and oxidation of LDL, thus inhibiting the formation of foam cells from macrophages, and plays an important role in the prevention of atherosclerosis. Proper modulation of its activity can be very useful for management of disorders of metabolic diseases.

Sfrp5 is among one of the AAKs which inhibits endothelial dysfunction, arterial stiffness and exhibits atheroprotective activity. CTRPs are the paralogs of adiponectin, and some members of CTRPs enhance insulin sensitivity and glucose metabolism. These members of CTRPs improve mitochondrial dysfunction, inhibit platelet activation and aggregations thereby reducing the risk of CAD thus preventing MI and stroke. They enhance the uptake of glucose by adipocytes thus conferring glucose homeostasis and also enhance cardiomyocyte survival and reduce fibrosis.

If properly designed and delivered, AAKs can represent a novel approach for anti-obesity, insulin sensitizing agents and anti-atherogenic therapies. For now, we can say that though novel and efficacious, adipokines still need to undergo considerable research for clinical safety and efficacy before we can see them in the market. At last we conclude that the diverse action of AAks has gained the attention of prominent researchers across the world and in future we may expect the use of these AAks as therapeutic agents for the metabolic disorders and its associated comorbidities.

## Data Availability

All data available in this article wherever applicable are collected from published articles and were cited. Figure 1 has been reproduced with due permission from the author (doi: 10.3389/fendo.2013.00071. eCollection 2013, From journal “Frontiers of Endocrinology” entitled “Adipokines mediate inflammation and insulin resistance” reference 86).

## Abbreviations

AAK: Anti-inflammatory adipokines
ABC: ATP-binding cassette
AN: Anorexia nervosa
ANGPTL4: Angiopoietin-like 4
AP-1: Activator protein-1
AT: Adipose tissue
AT: Adipose tissue
ATF6: Activating transcription factor 6
ATM: Adipose tissue macrophages
BAT: Brown adipose tissue
BMI: Body mass index
CACT: Carnitine acylcarnitine translocase
CAD: Coronary artery disease
CAMs: Cellular adhesion molecules
CDC: Centers for Disease Control and Prevention
CHD: Coronary heart disease
CMD: Cardiometabolic disease
CNS: Central nervous system
CoA: Coenzyme A
COX-2: Cyclooxygenase-2
CPT1: Carnitine palmitoyltransferase 1
CR: Caloric restriction
CTRP: C1q/TNF-related protein
CVD: Cardiovascular disease
DM: Diabetes mellitus
EAT: Epicardial adipose tissue
ECM: Extracellular matrix
ER: Endoplasmic reticulum
FA: Fatty acids
FABP: Fatty acid-binding protein
FAT/CD36: Fatty acid translocase CD36
FATP1: Fatty acid transporter protein 1
FFA: Free fatty acids
HAN: Hypertrophic adipocyte necrosis
HDL-C: High-density lipid cholesterol
HIF1-α: Hypoxia-inducible factor 1α
HMW: High molecular weight
HUVECs: Human umbilical vein endothelial cells
ICAM-1: Intracellular adhesion molecule
IFN-γ: Interferon gamma
IKK-β: Inhibitor of kinase-β
IL-10: Interleukin-10
IMTG: Intramuscular triacylglycerol
iNOS: Nitric oxide synthase
IRE-1: Inositol-requiring enzyme 1
IRS1: Insulin Receptor Substrate 1
JAK2: Janus kinase 2
JNK: C-Jun amino-terminal kinase
LCFA: Long-chain FA
LCFAT: Long-chain fatty acid transporter
LMW: Low molecular weight
LOX-1: Low density lipoprotein receptor-1
LRb: Leptin receptor b
LXR: Liver X receptor
MAPK: Mitogen-activated protein kinase
MARCO: Macrophage receptor with collagenous structure
MAT: Bone marrow adipose tissue
MCP-1: Monocyte chemoattractant protein-1
MI: Myocardial infarction
MIF: Macrophage migration inhibition factor
MMP-2: Matrix metallopeptidase 2
NAMPT: Nicotinamide phosphoribosyltransferase
NFkB: Nuclear factor kappa-B
NK: Natural killer cells,
NKT: Type-1 natural killer
ob: Obese gene
oxLDL: Oxidized low density lipid
OxPhos: Oxidative phosphorylation
PAK: Pro-inflammatory adipokines
PERK: PKR-like endoplasmic reticulum kinase
PI3-AKT: Phosphatidylinositol 3-kinase
PKB: Protein kinase B
PPAR: Peroxisome proliferator-activated receptor
RE: Redox environment
ROS: Reactive oxygen species
SAT: Subcutaneous adipose tissue
Sfrp5: Secreted frizzled-related protein 5
SOCS: Suppressor of cytokine signaling
SREBP-1c: Sterol regulatory element-binding protein 1c
STAT3: Signal transducer and activator of transcription 3
T2DM: Type 2 diabetes mellitus
TAG: Triacylglycerol
TCA: Tricarboxylic acid
TG: Triglyceride
TGF-β: Transforming growth factor beta
TH2: T helper 2
TLR: Toll-like receptors
TNF-α: Tumor necrosis factor-α
Treg: T regulatory cells
UPR: Unfolded protein response
VAT: Vascular adipose tissue
VCAM-1: Vascular cell adhesion molecule-1
VEGF: Vascular endothelial growth factors
VLDL: Very low density lipid
WAT: White adipose tissue
WHO: World Health Organization
